# Supplementary material for: Improved prediction of bacterial CRISPRi guide efficiency from depletion screens through mixed-effect machine learning and data integration
Source: Genome Biol. 2024 Jan 11;25:13. doi: 10.1186/s13059-023-03153-y (PMC10782694; doi:10.1186/s13059-023-03153-y)
Supplement: Supplementary file 1 — Additional file 1: Figure S1. Illustration of the genomic and sequence features used. Figure S2. Comparison of guide depletion across datasets. Figure S3. Spearman correlation of 10-fold cross-validation of models trained with one or mixed datasets. Figure S4. Data integration for retrained Pasteur and deep learning models. Figure S5. Interaction between distance features and whether targeting gene is the first gene in operon. Figure S6. Independent low-throughput validation of model performance. Figure S7. Additional figures related to the saturating screen of purine biosynthesis genes. Figure S8. Model performance of deep learning approaches. Supplementary Note: Deep learning approaches do not improve prediction performance. [file 13059_2023_3153_MOESM1_ESM.pdf]

Additional File 1: Improved prediction of bacterial CRISPRi guide efficiency from depletion screens through mixed-effect machine learning and data integration

Yanying Yu[1], Sandra Gawlitt[1], Lisa Barros de Andrade e Sousa[2], Erinc Merdivan[2], Marie Piraud[2], Chase L. Beisel[1,3], Lars Barquist[1,3]

[1]Helmholtz Institute for RNA-based Infection Research (HIRI) / Helmholtz Centre for Infection Research (HZI), 97080 Würzburg, Germany.

[2]Helmholtz AI, Helmholtz Zentrum München, 85764 Neuherberg, Germany.

[3]Medical Faculty, University of Würzburg, 97080 Würzburg, Germany.

Supplementary Figures

Figure S1

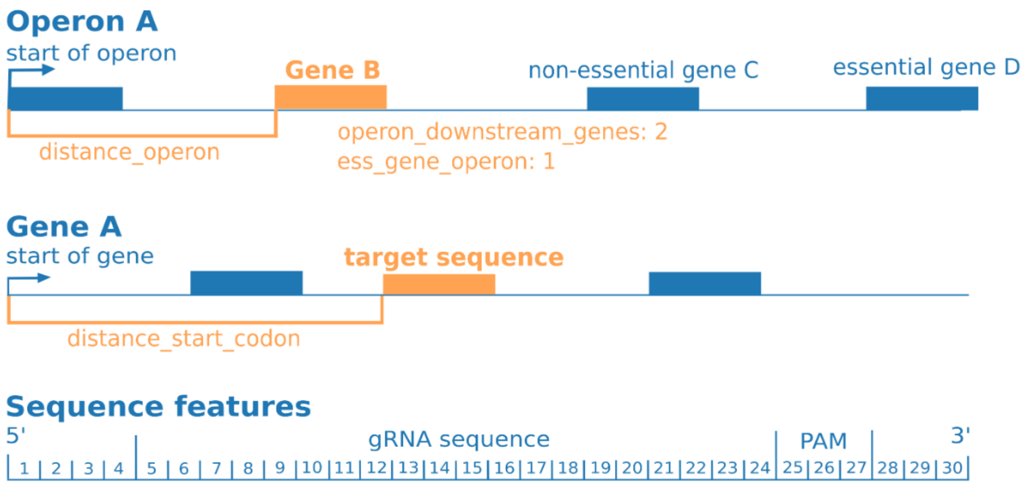

Figure S1: Illustration of the genomic and sequence features used, see also Table S1.

Figure S2

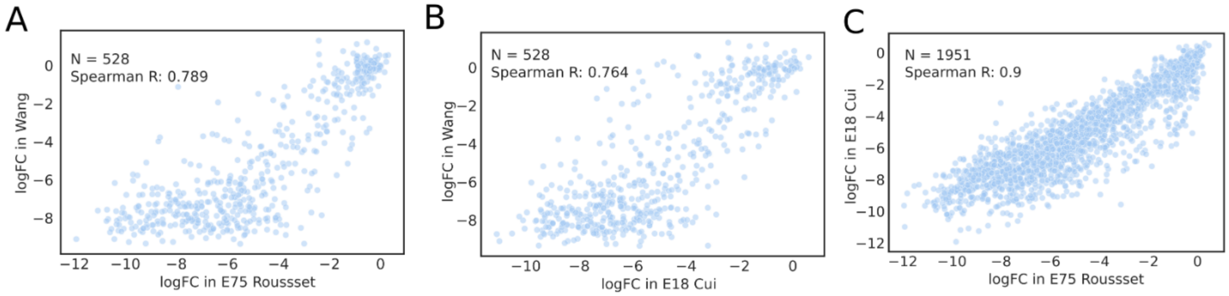

Figure S2: Comparison of guide depletion across datasets (**A**) The logFC of gRNAs in E75 Rousset plotted against that in Wang for shared gRNAs. (**B**) The logFC of gRNAs in E18 Cui was plotted against that in Wang for shared gRNAs. (**C**) The logFC of gRNAs in E18 Cui was plotted against that in E75 Rousset for overlapping gRNAs.

Figure S3

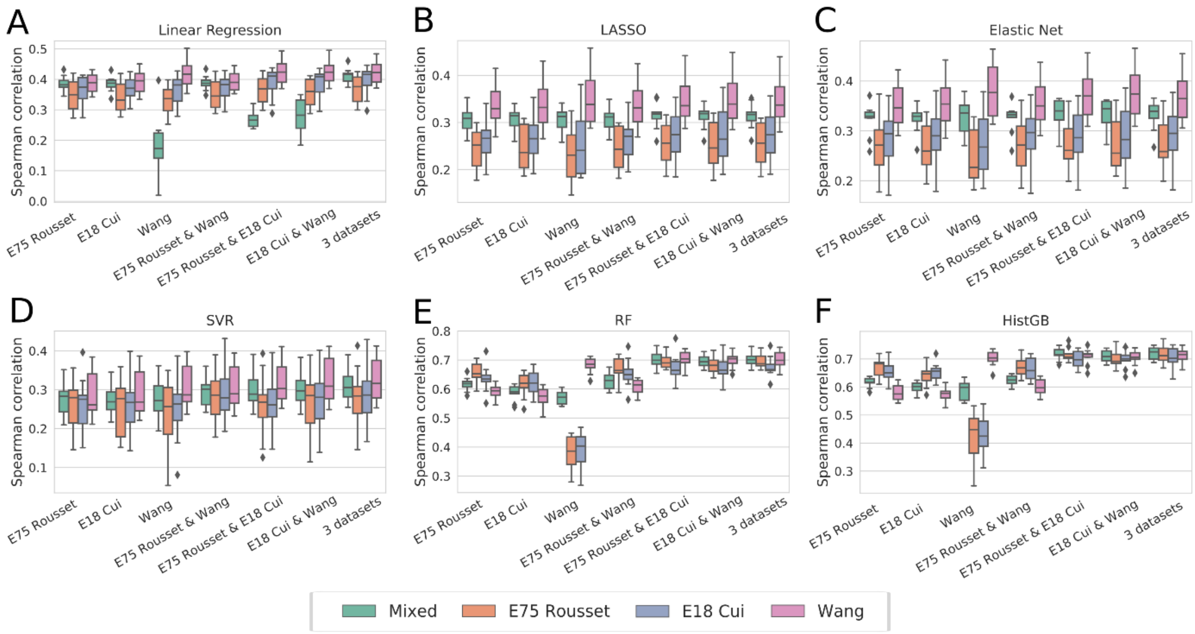

Figure S3: Spearman correlation of 10-fold cross-validation of models trained with one or mixed datasets. (A) linear regression, (B) LASSO, (C) Elastic net, (D) support vector regression (SVR), (E) Random forest (RF) regression, (F) Histogram-based gradient boosting regression.

Figure S4

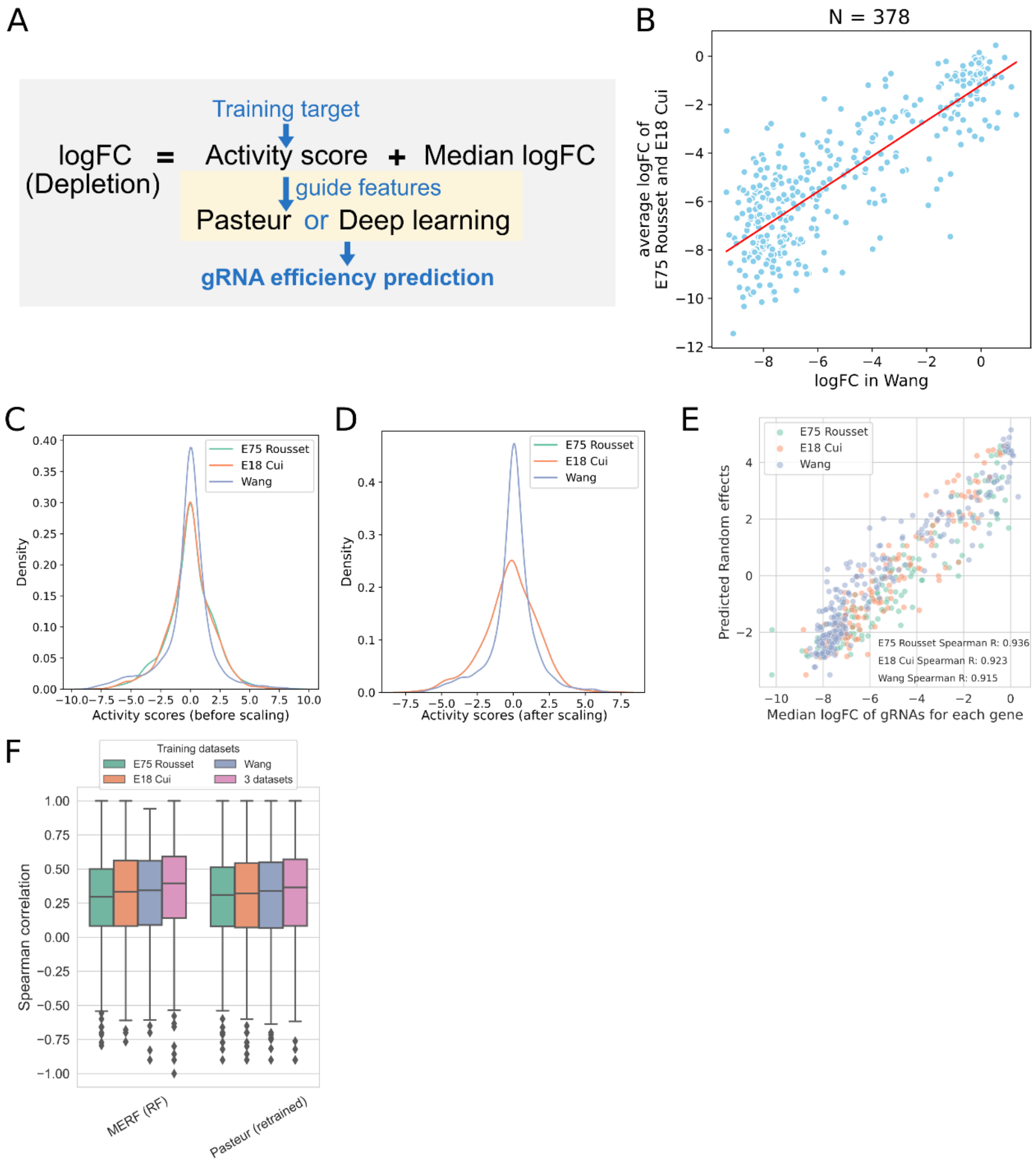

Figure S4: Data integration for retrained Pasteur and deep learning models. (**A**) The overview of retraining the Pasteur and deep learning models. We subtract the gene-wise median logFC from each gRNA depletion value upon data fusion to obtain the activity scores of each gRNA. The activity scores were used as training targets and the Pasteur model and deep learning models were trained with guide-specific features. (**B**) The logFC values in Wang were scaled based on a linear regression between the original logFC of Wang and the average logFC of E75 Rousset and E18 Cui for 378 overlapping gRNAs. The distributions of activity scores (**C**) with and (**D**) without scaling are shown. (**E**) Predicted

scores of the random effect model from MERF (y-axis) compared to the median logFC across gRNAs (x-axis) for each gene in each dataset. (**F**) Boxplots illustrating the distribution of Spearman correlations for guides in held-out genes during cross-validation.

Figure S5

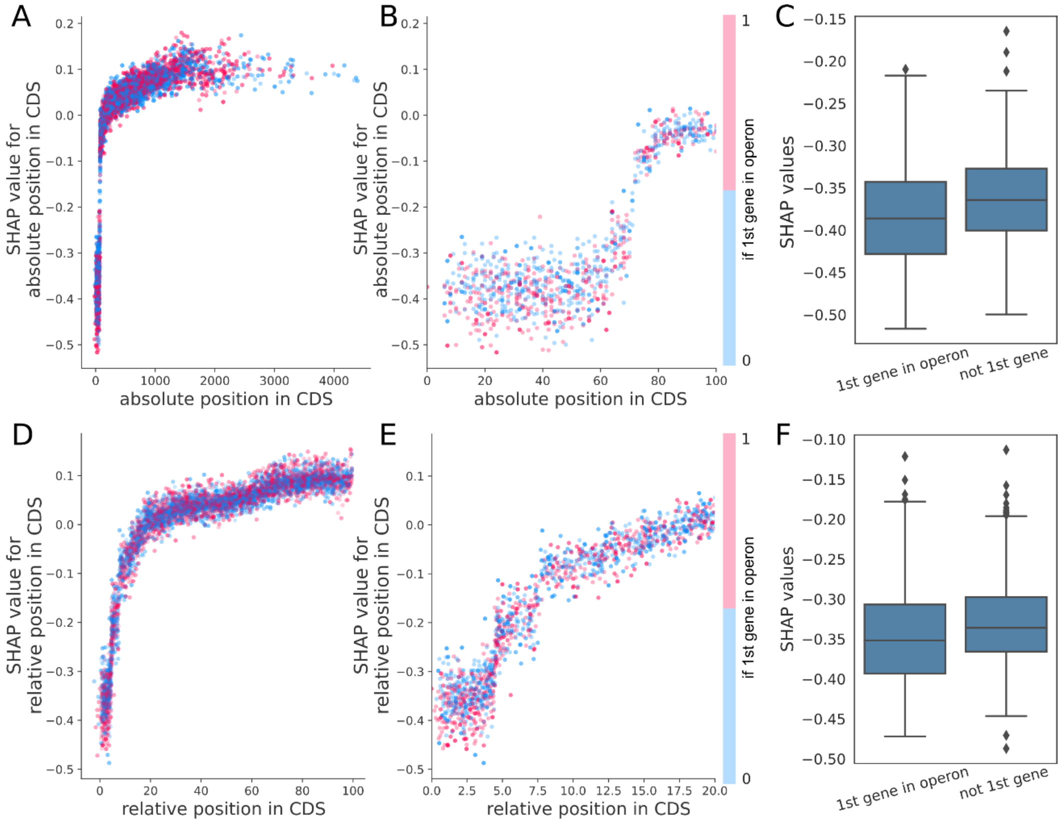

G

Model	Feature	Median Spearman Correlation Across held-out genes			
		**E75 Rousset**	**E18 Cui**	**Wang**	**Mixed**
**MERF**	with 1st gene in operon	**0.409**	0.400	**0.373**	**0.396**
	without 1st gene in operon	0.396	**0.409**	0.350	0.385

Figure S5: Interaction between distance features and whether targeting gene is the first gene in operon. (**A-B**) SHAP values for absolute position in CDS plotted against absolute position. The dots are colored based on whether the guide is targeting the 1st gene in operon. Red indicates the guide targets the 1st gene in operon, while blue indicates it is not. In **B**, the x-axis is limited to range 0-100. (**C**) SHAP values for absolute position in CDS of guides targeting within the first 70 bases. SHAP values are significantly lower for 1st genes in operons ($p = 2.13 \times 10^{-8}$, two-sided Mann-Whitney U test). (**D-E**) Similar to A-B but for relative position in CDS. In **E**, the x-axis is limited to range 0-20. (**F**) SHAP values for relative position in CDS of guides targeting relative position in CDS lower than 5%. SHAP values are significantly lower for 1st genes in operons ($p = 1.68 \times 10^{-5}$, two-sided Mann-Whitney U test). (**G**) Evaluating predictions of guide efficiency after removing gene effects for MERF models trained with or without the feature of whether the target gene is the 1st gene in operon, using the same cross-validation procedure as Figure 2B.

Figure S6

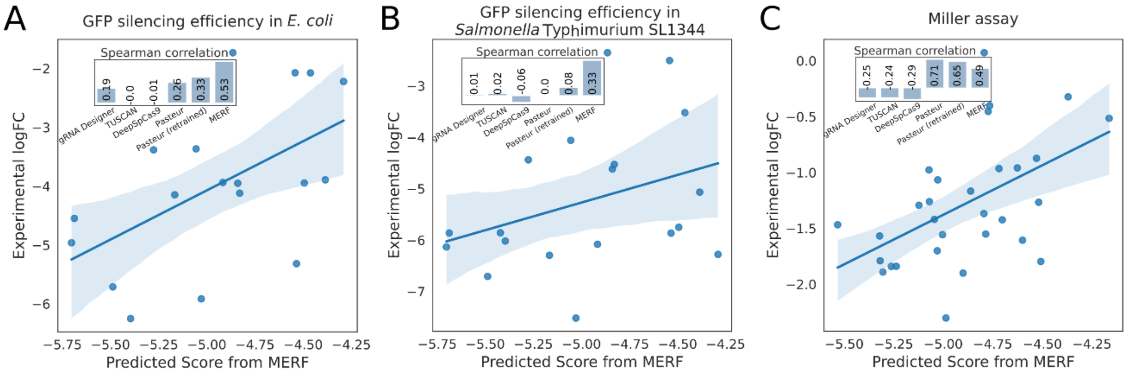

Figure S6: Independent low-throughput validation of model performance. The activity of 19 gRNAs targeting a plasmid-expressed deGFP gene was measured in (**A**) *E. coli* and in (**B**) *Salmonella* Typhimurium SL1344 using a flow cytometry-based assay. The measured activity compared to the control gRNA is plotted against the score predicted by the MERF model. The inset barplot illustrates Spearman correlations for six methods for predicting guide efficiency. (**C**) The activity of 30 gRNAs targeting lacZ was measured with a Miller assay by [1], plotted as in A and B.

Figure S7

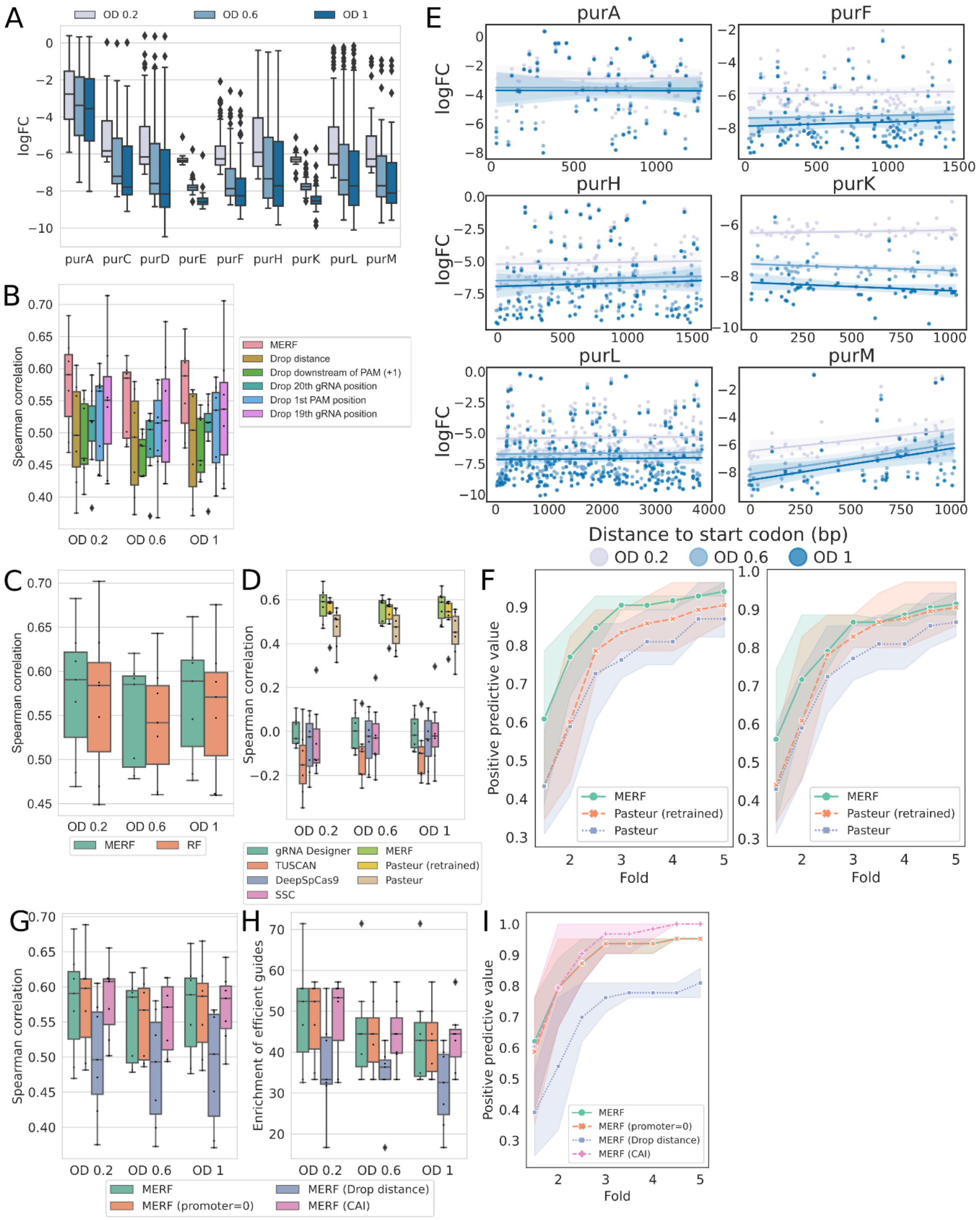

Figure S7: Additional figures related to the saturating screen of purine biosynthesis genes. (**A**) The distribution of experimental logFC at different time points for each gene. (**B**) Effects on validation

Spearman correlation of dropping important features identified by SHAP analysis (**Figure 3A**). Dropping either distance features or features related to important sequence positions leads to a clear decrease in model performance ($p < 0.001$ for all comparisons, paired t-test.) (**C**) Comparison of the MERF model to a random forest model trained on activity scores. The MERF model performs slightly, but not significantly ($p=0.33$, paired t-test) better than the standard random forest model. (**D**) Spearman correlations between the predicted scores and measured logFC across collected timepoints. (**E**) Measured logFCs for each guide as a function of distance to the start codon for the other 6 genes not shown in Figure 4E. (**F**) Positive predictive values of all gRNAs for each time point. The predicted positives are defined as (**left**) the top four and (**right**) the top five predicted gRNAs in each gene, while true positives are gRNAs with fold change within the N fold of the strongest depleted gRNA in each gene (N= 1.5 - 5 with a step of 0.5). (**G-I**) Performance on the purine screen of MERF models trained with the promoter feature set to 0 (promoter=0), without distance features (Drop distance), and with 4 instead of 9 gene features for the random-effect model (CAI value, gene length, gene GC content, and dataset). The calculation of Spearman correlation, enrichment of efficient guides, and positive predictive value are the same as Figure 4B-D.

Figure S8

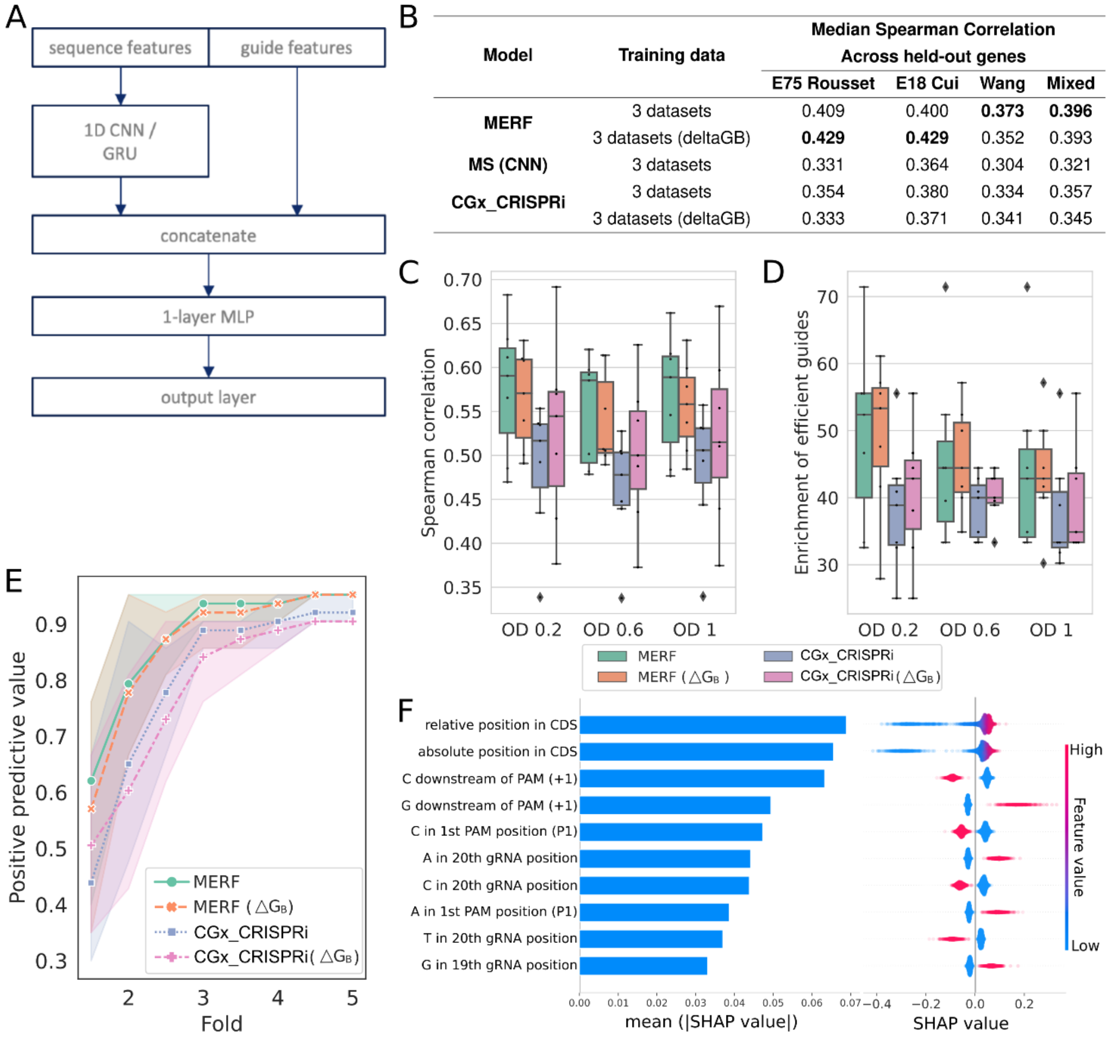

Model	Training data	Median Spearman Correlation Across held-out genes			
		E75 Rousset	E18 Cui	Wang	Mixed
MERF	3 datasets	0.409	0.400	**0.373**	**0.396**
	3 datasets (deltaGB)	**0.429**	**0.429**	0.352	0.393
MS (CNN)	3 datasets	0.331	0.364	0.304	0.321
CGx_CRISPRi	3 datasets	0.354	0.380	0.334	0.357
	3 datasets (deltaGB)	0.333	0.371	0.341	0.345

Figure S8: Model performance of deep learning approaches. (**A**) Architectures of the applied deep learning models. Guide features refer to guide-specific features apart from sequence features. MLP: multilayer perceptron. (**B**) Evaluating predictions of guide efficiency after removing gene effects. Spearman correlations between predictions and measured logFC for held-out genes. Genes were held out in 10-fold cross-validation, and the reported median Spearman correlation was calculated across all held-out genes. When $\triangle G_B$ is specified, the MERF or CGx_CRISPRi models were trained with $\triangle G_B$ instead of the original four thermodynamic features. (**C-E**) Performance on the purine screen of MERF and CGx_CRISPRi models. When $\triangle G_B$ is specified, the MERF or CGx_CRISPRi models were trained with $\triangle G_B$ instead of the original four thermodynamic features. The calculation of Spearman correlation, enrichment of efficient guides, and positive predictive value are the same as Figure 4B-D. (**F**) SHAP values for the top 10 features from MERF model trained with $\triangle G_B$. Global feature importance is given by

the mean absolute SHAP value (left), while the beeswarm plot (right) illustrates feature importance for each guide prediction.

Supplementary Note: Deep learning approaches do not improve prediction performance.

Given that we saw better performance with tree-based methods over linear regression, we asked if model complexity was a limiting factor in prediction. Deep learning approaches have been applied to predicting guide efficiency for a number of CRISPR technologies [2–6]. Considering this, we asked if deep learning models would also improve performance in predicting gRNA efficiency for CRISPRi in bacteria. As a representative architecture, we implemented a one-dimensional convolutional neural network (CNN), which runs a series of kernel filters across the sequence to extract local features. As a second model representative of current deep learning approaches, we reimplemented a state-of-the-art deep learning architecture based on the CGx architecture used by CRISPRon [6] for Cas9 genome editing in eukaryotes, only trained using our CRISPRi data and using our feature set. Both models were trained using scaled data as done for re-training the Pasteur model (see Data integration for retrained Pasteur and deep learning models, **Methods**).

For our custom CNN architecture, we used the convolutional layers to extract sequence features before concatenating them to the rest of our guide feature set (**Figure S8A**). This concatenated feature set was then fed through a fully connected 4-layer multilayer perceptron (MLP) for regression using MS values for guide efficiency. Both the custom CNN and CGx_CRISPRi models exhibited lower Spearman correlations as compared to our previously trained random forest models when tested on held-out gene sets (**Figure S8B**; Table S7; CNN ρ=0.321, CGx_CRISPRi ρ=0.357, vs. MERF ρ=0.396). As one major difference between our CGx_CRISPRi implementation and that of CRISPRon is in the use of the $\triangle G_b$ parameter that integrates a complete thermodynamic model of Cas9 binding [7], we additionally tested substituting this for our simpler thermodynamic features in both CGx_CRIPSRi and our MERF model, leading to no clear improvement in prediction performance (**Figure S8BCD&E**). These results show that conventional machine learning approaches can

outperform deep learning architectures and suggest that data may currently be limiting for more complex machine learning approaches, though it remains possible that specialized architectures may improve on the approaches implemented here.

References

1. Calvo-Villamañán A, Ng JW, Planel R, Ménager H, Chen A, Cui L, et al. On-target activity predictions enable improved CRISPR-dCas9 screens in bacteria. Nucleic Acids Res. 2020. doi:10.1093/nar/gkaa294

2. Chuai G, Ma H, Yan J, Chen M, Hong N, Xue D, et al. DeepCRISPR: optimized CRISPR guide RNA design by deep learning. Genome Biol. 2018;19: 80.

3. Kim HK, Min S, Song M, Jung S, Choi JW, Kim Y, et al. Deep learning improves prediction of CRISPR-Cpf1 guide RNA activity. Nat Biotechnol. 2018;36: 239–241.

4. Kim HK, Kim Y, Lee S, Min S, Bae JY, Choi JW, et al. SpCas9 activity prediction by DeepSpCas9, a deep learning-based model with high generalization performance. Sci Adv. 2019;5: eaax9249.

5. Wang D, Zhang C, Wang B, Li B, Wang Q, Liu D, et al. Optimized CRISPR guide RNA design for two high-fidelity Cas9 variants by deep learning. Nat Commun. 2019;10: 4284.

6. Xiang X, Corsi GI, Anthon C, Qu K, Pan X, Liang X, et al. Enhancing CRISPR-Cas9 gRNA efficiency prediction by data integration and deep learning. Nat Commun. 2021;12: 3238.

7. Alkan F, Wenzel A, Anthon C, Havgaard JH, Gorodkin J. CRISPR-Cas9 off-targeting assessment with nucleic acid duplex energy parameters. Genome Biol. 2018;19: 177.
