# Supplementary material for: Improved prediction of bacterial CRISPRi guide efficiency from depletion screens through mixed-effect machine learning and data integration
Source: Genome Biol. 2024 Jan 11;25:13. doi: 10.1186/s13059-023-03153-y (PMC10782694; doi:10.1186/s13059-023-03153-y)
Supplement: Supplementary file 3 — Additional file 3. Review history. [file 13059_2023_3153_MOESM3_ESM.docx]

Review History

**First round of review**

**Reviewer 1**

**Were you able to assess all statistics in the manuscript, including the appropriateness of statistical tests used?** No.

**Were you able to directly test the methods?** Yes.

**Comments to author:**

In this manuscript, the authors used several kinds of features to develop CRISPRi guide efficiency prediction models. They also found that the combination of a mixed-effect random forest regression model with gene-specific features achieved good performance. They developed a web server for CRISPRi guide efficiency prediction. The idea of this manuscript sounds good, but the results don't seem very good. The main question is it is not convincing whether their model surpassed the previous model if they were train using the same dataset. Besides, systematic benchmark to validate the "state-of-the-art" performance of this model is required.

1. I don't quite understand why the authors used gene ID as a predictor, and I cannot find any description how they used it in the Methods section.

2. The authors should convince that the models developed by them were better than the previous ones, so more comparisons should be presented, for example, in Figure 2B. Moreover, I don't think the Pasteur model is the only one for comparison. The SSC scoring method (http://www.genome.org/cgi/doi/10.1101/gr.191452.115) is capable of scoring the CRISPRi efficiency.

3. The benchmarking scenarios are far from satisfaction. Firstly, the authors used gene expression, which is cell type-specific, to develop the models, while the benchmarking datasets were from the same cell type. Secondly, they used a model trained on 3 datasets to compare the Pasteur model, which was trained on only one of the three datasets.

4. One of the sections was entitled "Deep learning approaches do not improve prediction performance". I don't think the authors can draw such a conclusion according to their simple comparison. The CNN model they used in this section was too simple to represent deep learning models.

5. Figure 4 A-B: The authors used 19 gRNAs to validate their models. As we can see, the data points are sparse and the results are not convincing. Also, as is shown in Figure 4B, the MERF model do not surpass the Pasteur model.

6. Figure 4D: The Pasteur model was trained on R75 dataset. According to the results, the MERF(R75) model do not surpass the Pasteur model.

**Reviewer 2**

**Were you able to assess all statistics in the manuscript, including the appropriateness of statistical tests used?** No.

**Were you able to directly test the methods?** Yes.

**Comments to author:**

Yu et al presents a new tool for prediction of loss of function of CRISPRi-Cas9 gRNAs. In the paper Yu et al first use publicly available data to construct a data set of changes in expression for an "essential genes" collection as result of employed gRNAs. Subsequently the authors generate their own data to make an independent data set.

In the first part the author conducts a 10-fold cross-validation analysis using these expression changes as output and the gRNA as one-hot encoded with additional features subsequently added. It remains unclear precisely what range the values of the output lie in and it remain unclear how much context of the gRNA is included in the one-hot encoding. Upon inspecting the supplementary excel file it seems that 30 nt is used of the gRNAs in the part concerning the 10-fold cross-validation, while not context is provided the authors own data. The authors need to extend their "Training datasets" subsection in Methods to "Datasets" and add concise descriptions (including the above mention unclear points) of all input and output data complemented by pointers from the Results section.

The authors do not clearly mention how the 10 folds (l 57-58) have been made (A pointer to p 32 would have been helpful). They do not describe the overlap between the 10 folds. If there are near identical gRNAs in any of the folds. it makes no sense to report the performance over the 10 folds (the average I assume). The authors should report on the overlap of the gRNA (e.g. hamming distance) in the validation sets and show that this strategy do not lead to near identical gRNAs in different validation sets and thereby overfitting. If there are paralogue genes in different validation sets this could be a potential issue.

In relation to this, the size and selection of held out dataset for testing of 10-fold cross validation seem not to be described anywhere. What are the sizes of the validation sets and which genes AND gRNAs are in each of these sets. The authors should not only present this, but also justify that their specific split is representative, i.e., could the performance change drastically if they ended up with another split by the same criteria?

It is unclear which gRNAs have been used for the independent test as sequences in both directions are given in Table S16. Also appear to be missing are sequence context of the targets and other inputs used for the model evaluation of these guides. The authors should clarify this.

It is also unclear why both strands seems to targeted, especially if NGG pam is needed

SG317 SG_sgRNA_degfp_ML1_F tagtAACGCCAGTGAAAAGCTCCA

SG318 SG_sgRNA_degfp_ML1_R aaacTGGAGCTTTTCACTGGCGTT

After some efforts, the upper case sequence appears to be the guide with _F matching the synthetic GFP gene. The test set is from Table S16, training data is from github https://github.com/BarquistLab/CRISPRi_guide_efficiency_bacteria.

This needs to be explained more carefully in the manuscript. Also it would be helpful if the authors explicitly point to github in this context.

Upon diving in to the data a bit of similarity to between training data and test data:

test training data from github

16 CGCTCCTGGACGTAGCCTTC CGCTTCTGTTCGTAGCTTTC

16 CGTCGCCGTCCAGCTCGACC TGTCGCCGCCCAGCTCGATT

16 CTTCAGGGTCAGCTTGCCGT CTTCCGGTGCAGCTTGCAGT

17 GACGTAGCCTTCGGGCATGG GACATAGCCTTCGGGAATAG

The authors are probably ok in overlap between test and training set, but it should be reported. What remain is that the authors justify why gRNAs on these genes are representative to serve as independent evaluation.

The authors state that they "re-implement" CRISPRon. Since this is not actual CRISPRon method and since are profound differences (see the following) it is misleading and confusing to call it CRISPRon. The authors should call it some else and then when first time mentioned state precisely which parts are inspired by CRISPRon and which parts are not.

In the scan of meta parameters for CNN and "CRISPRon" the authors should mention these, including batch size and learning rates. They should also point what is the same and difference for CRISPRon.

It seems that the authors re-implementation of CRISPRon is not exact if the do not use the same architecture is as CRISPRon. The authors state (p15, 26-30): "we reimplemented and tested the state-of-the-art deep learning architecture used for predicting Cas9 gene editing efficiency by CRISPRon (Xiang et al., 2021), only trained using our CRISPRi data." However, they do not present how the resulting binding energy Delta GB is fed into the network. The authors need to explain this carefully.

Furthermore, it seems that the Yu et al, do not even have Delta GB as a single parameter and that "only" a hybridization energy and a gRNA spacer self-folding is included, but separately. And hence the authors seem to ignore the DNA opening energy. The authors should elaborate on what they precisely do and explain why their re-implementation ignores the opening energy.

The authors calculate DNA-gRNA hybridization energy from the Vienna package(p6, 11-12). As I understand Xiang their DNA-gRNA hybridization energy includes weights on each stacked pair corresponding to influence of the protein. This is apparently not the case for the authors re-implementation. Again, the authors need to elaborate and argue why their version of hybridization energy is more suitable.

For the SHAP analysis, I wonder if the authors compared to the one by Xiang et al, which showed that Delta GB is the most important feature SHAP and Gini importance. Can the authors comment on this?

For the SHAP analysis on Fig 3A. The +1 PAM favoring efficient gRNAs was recently described by Corsi et al., 2022, Nat comm and the authors comment on that this already is known.

There are what appear as as weird features in Table S3 including:

features Random Forest model trained with E75Rousset

gene_expression_max 0.782893970297067

ess_gene_operon 0.490418806310321

gene_GC_content 0.293801473999851

gene_expression_min 0.194561228228971

gene_length 0.237718945757704

geneid 0.174114175877214

distance_operon 0.151180464424676

dist_start_codon 0.147285478273062

dist_start_codon_perc 0.120338853153561

dataset 0

Although many of these seemingly have been removed to subsequent SHAP tables (S7 and S8) they should never have been used in the first place.

More elaborate:

I am aware that the authors define what they refer to as call minimum and maximum expression values (p6, 34-37), but it is unclear precisely how they are defined.

Do these minimum and maximum expression values even make sense when making prediction on an external data, where e.g. the expression is in another range? The authors should elaborate.

I am also concerned of the usage of the absolute position in the gene can create biases, since disruption closer to the 5' end will more potentially favor shorter genes and learn to distinguish short from long genes. The authors also show such bias in Fig 4E. Given the authors work with a small genome (compared to Eurakyote) the effect might be minor. Nonetheless and in spite that it sadly has been used on several instances in the literature, this feature should not be used. The authors remove this feature then report the performance again.

Similar concerns can be raised for "gene length", "distance_operon" etc…

What is potentially even bigger concerns is the authors use of "geneid" as a feature. Why would one what to label different gRNAs by the gene they come from? How would the authors in any meaningful way instruct a user that comes with an external data set, or simply just want to make a prediction on a specific gRNA?

Similar concern for "dataset", which I do not see clearly defined.

Minor:

When clicking on references I end up at unreadable links.

**Authors Response**

**Point-by-point responses to the reviewers’ comments:**

We would like to thank both reviewers for their thorough review of our manuscript and for their in-depth comments. In response we have substantially revised our manuscript to clarify our two main findings: 1) that effects from targeted genes dominate over the effects of guide efficiency in depletion screens, which needs to be removed to produce accurate predictions of guide efficiency, and 2) that integrating multiple data sets is important to increase prediction accuracy given currently available data.

To further clarify point 1, we have introduced a new figure panel (2A) which illustrates our approach and further described it in the text, as this appeared to be unclear to both reviewers. As presented in figure 1A, the data that we work with is depletion data from competitive growth experiments between strains carrying gRNAs targeting dCas9 to silence bacterial genes. This means the logFC only indirectly reports on guide efficiency, as the primary effect is from silencing of the targeted gene.

To extract guide efficiency from these indirect measurements, we train a mixed-effect random forest that includes a linear random effect model for each gene that uses gene features (e.g. expression, gene length, GC content), and a fixed-effect random forest that uses guide features (e.g. sequence features, thermodynamic features, distance features).

Importantly, the purpose of the random effect model is solely to remove the effect of gene features, and the inclusion of gene ID serves as a cluster ID to group guides arising from the same gene. Gene ID itself is not used a feature in the final model, and we have removed gene ID from our depletion models to avoid confusion. For all prediction tasks, only the fixed-effect guide efficiency model is used; hence, gene features like expression level and gene ID are not required to use the model.

We have also removed discussion of models trained on scaled data to focus on comparing our fixed-effect model to the previously published Pasteur model and a new, retrained version of Pasteur that we have implemented ourselves using the authors’ code. During revision we also discovered that removal of dinucleotide features improved our model performance in cross-validation and simplified interpretation of feature importance. Our results have been updated to reflect this. To reduce the length of our manuscript and further highlight our main findings, we have moved discussion of our scaling normalization to the methods only, and of deep learning models to a supplementary note referenced in the text.

All changes to the text have been highlighted in red in the submitted revised version.

----------------------------------------------------------------------------

*Reviewer #1: In this manuscript, the authors used several kinds of features to develop CRISPRi guide efficiency prediction models. They also found that the combination of® a mixed-effect random forest regression model with gene-specific features achieved good performance. They developed a web server for CRISPRi guide efficiency prediction. The idea of this manuscript sounds good, but the results don't seem very good. The main question is it is not convincing whether their model surpassed the previous model if they were train using the same dataset. Besides, systematic benchmark to validate the "state-of-the-art" performance of this model is required.*

*1. I don't quite understand why the authors used gene ID as a predictor, and I cannot find any description how they used it in the Methods section.*

Response:

This was previously described in the Methods on page 31. Gene ID is used as a random effect cluster ID to group guides targeting the same gene during mixed effect modeling, so that the effect of the targeted gene may be removed. Gene ID is not directly used as a predictor in the mixed effect model. We have also clarified our use of gene ID in the main text. We have removed gene ID as a predictor in our depletion model in Figure 1 to avoid confusion.

----------------------------------------------------------------------------

*2. The authors should convince that the models developed by them were better than the previous ones, so more comparisons should be presented, for example, in Figure 2B. Moreover, I don't think the Pasteur model is the only one for comparison. The SSC scoring method (http://www.genome.org/cgi/doi/10.1101/gr.191452.115) is capable of scoring the CRISPRi efficiency.*

Response:

To our knowledge, the Pasteur model remains the only published method for predicting CRISPRi guide efficiency in bacteria. On the reviewer’s suggestion, we have tested SSC on our validation data set. SSC’s performance was uniformly poor, with Spearman correlations close to 0. Given the poor performance and that SSC was not trained for bacterial CRISPRi, we have chosen not to include this result in our main benchmark comparison in Figure 4, but we do include SSC in Figure S7D along with several other methods for predicting CRISPR gene editing efficiency in eukaryotes that all have similarly poor performance on bacterial CRISPRi.

To further support our claims regarding the performance of our fixed effect model, we have devised two additional metrics using our saturating screen meant to mimic guide selection that might occur in the context of designing a genome-wide screen or silencing a particular gene-of-interest. In both cases, the MERF model outperforms the published Pasteur model by a wide margin (Figure 2B, Figure 4BC&D).

----------------------------------------------------------------------------

*3. The benchmarking scenarios are far from satisfaction. Firstly, the authors used gene expression, which is cell type-specific, to develop the models, while the benchmarking datasets were from the same cell type.*

Response:

We would note that the publicly available depletion data we used for training was grown in rich media (Lysogeny Broth), while our test data set was from growth in M9 minimal media. It is true that the expression data that we included was also from growth in MOPS minimal media, which may lead to similar expression profiles as M9.

However, none of the pur genes in our test data set were included in our initial training data, as the pur genes are only essential in minimal media. Thus, the model has never seen expression values (or any other features) for any of these genes prior to the validation task. We have additionally tested using the codon adaptation index (CAI), a proxy for gene expression based solely on codon usage patterns in the genome sequence, as an alternative to including actual expression data for training. We achieve similar performance using CAI in place of expression data (Figure S7EF&G), further indicating that the performance of our model is not dependent on having access to expression information specific to assay conditions.

----------------------------------------------------------------------------

*Secondly, they used a model trained on 3 datasets to compare the Pasteur model, which was trained on only one of the three datasets.*

Response:

A major theme in our manuscript is that integrating multiple datasets improves model prediction without degrading performance on any particular dataset, mentioned in both the title and discussed extensively in the section “Data fusion improves prediction performance”. We believe that this is a valuable contribution in itself, particularly now that we have shown that this generalizes to the Pasteur model as well.

We also would like to note that the final model consists of both the underlying regressor and its parameters learned from the data. It is common in the field to train predictive models on new datasets as they become available and compare these to older methods trained on different data. In fact, it would not be possible to achieve the accuracies reported by many of these new methods (e.g. some deep learning approaches) when training on the smaller datasets earlier methods were trained on. For recent examples where similar comparisons have been made, please see:

Figure 3 of Kim et al., “SpCas9 activity prediction by DeepSpCas9, a deep learning–based model with high generalization performance”, Science Advances 2019

Figure 2B of Xiang et al., “Enhancing CRISPR-Cas9 gRNA efficiency prediction by data integration and deep learning”, Nature Communications 2021

At the reviewer’s suggestion, we have retrained the Pasteur model using our expanded dataset. This was not trivial and would likely be beyond what could reasonably be expected of a typical end user, requiring application of a scaling normalization method to correct for batch effects as described in the methods section, and modification of the original source code. In keeping with our earlier results, we find that training on multiple datasets improves performance for the Pasteur model as well. Our fixed effect random forest derived from our MERF model retains superior performance as measured by Spearman correlation across genes (Figure 4B), and now additionally shown for enrichment of active guides in the best scoring 20% of guides (Figure 4C), and positive predictive value for the top 3, 4, or 5 predicted most active guides (Figure 4D).

----------------------------------------------------------------------------

*4. One of the sections was entitled "Deep learning approaches do not improve prediction performance". I don't think the authors can draw such a conclusion according to their simple comparison. The CNN model they used in this section was too simple to represent deep learning models.*

Response:

We tested two deep learning models: a one-dimension CNN and a second model based on the CRISPRon model introduced by Xiang et al 2021, and neither had better performance than the MERF-derived random forest (Figure S8). As previously stated in the text, we believe that data availability is likely to be the limiting factor currently. Regardless, we agree that this is a fairly minor point and have moved these results to a supplementary note and additionally note that it is possible other deep learning architectures may improve performance.

----------------------------------------------------------------------------

*5. Figure 4 A-B: The authors used 19 gRNAs to validate their models. As we can see, the data points are sparse and the results are not convincing. Also, as is shown in Figure 4B, the MERF model do not surpass the Pasteur model.*

Response:

We fully agree with the reviewer that the data previously presented in Figures 4A&B is not convincing, and it was primarily meant to motivate our validation screen using 750 guide RNAs targeting 9 genes not present in our training data (previously Figures 4 C&D). We have now moved the low-throughput data to supplementary information and only point to it in the main text with the comment that the data is too sparse to justify strong conclusions.

----------------------------------------------------------------------------

*6. Figure 4D: The Pasteur model was trained on R75 dataset. According to the results, the MERF(R75) model do not surpass the Pasteur model.*

*Response:*

This is true, though the differences in Spearman correlation between the original Pasteur model and our model trained only on R75 are less pronounced now that we have revised our feature set, and the MERF (R75) performs better than the Pasteur R75 on enriching for efficient gRNAs and in the positive predictive value for the 3 best scoring gRNAs(Table S14).

However, our goal in this work was to produce the best possible predictor of CRISPRi guide efficiency. This predictor includes both the regressor used, as well as the parameters derived from training. An important point of our work was that data integration improves performance and generalization without degrading performance on individual datasets in cross-validation. This is not completely obvious, as the datasets come from different labs with differences in the growth protocols and expression constructs, and there could in principle be factors important for prediction that are specific to each dataset.

It is clear from the cross-validation results in Figure 2B and validation results in Figure 4BC&D that our random forest model surpasses the previous state-of-the-art, which was the Pasteur model trained on the R75 dataset. Retraining the Pasteur model on fused datasets also substantially improves performance, illustrating our findings regarding the importance of data fusion. The retrained Pasteur model does not exceed the performance of our MERF model, further showing that our choice of regressor and features also improves performance.

----------------------------------------------------------------------------

*Reviewer #2: Yu et al presents a new tool for prediction of loss of function of CRISPRi-Cas9 gRNAs. In the paper Yu et al first use publicly available data to construct a data set of changes in expression for an "essential genes" collection as result of employed gRNAs. Subsequently the authors generate their own data to make an independent data set.*

*In the first part the author conducts a 10-fold cross-validation analysis using these expression changes as output and the gRNA as one-hot encoded with additional features subsequently added.*

Response:

We would like to clarify that we do not have direct access to changes in expression resulting from CRISPRi targeting. The data sets we work with only record depletion within pooled fitness screens, which is a combination of the fitness effect due to (partial) silencing of the target with the strength of silencing induced by the gRNA (i.e. guide efficiency), illustrated in Figure 1A. This was the primary motivation in developing our mixed-effect modeling approach which can separate these two effects and provide us with a predictor of guide efficiency.

----------------------------------------------------------------------------

*It remains unclear precisely what range the values of the output lie in …*

Response:

Our output values from the MERF model fall in the range of -12 to +2, the same range as observed in the training data. Due to guide efficiency being only indirectly measured, predicted values from the fixed-effect model do not have a straight-forward interpretation in terms of the original depletion values. They are the estimated residual log2 fold change in gRNA abundance after removing the estimated effect of the targeted gene on gRNA abundance, and mostly fall in the range of -7 to -2.5. We have added this to the methods section on page 31.

----------------------------------------------------------------------------

*…and it remain unclear how much context of the gRNA is included in the one-hot encoding. Upon inspecting the supplementary excel file it seems that 30 nt is used of the gRNAs in the part concerning the 10-fold cross-validation, while not context is provided the authors own data. The authors need to extend their "Training datasets" subsection in Methods to "Datasets" and add concise descriptions (including the above mention unclear points) of all input and output data complemented by pointers from the Results section.*

Response:

We have used the same sequence context (30 nt) in all of our work, as previously shown in figure S1 and described in Table S1 “input_feature_description”. For the purine screen guides, the context was included in Table S13 (previously S12) “purine_screen_guides”. We have attempted to clarify this in the Methods and now explicitly point to these descriptions.

----------------------------------------------------------------------------

*The authors do not clearly mention how the 10 folds (l 57-58) have been made (A pointer to p 32 would have been helpful). They do not describe the overlap between the 10 folds. If there are near identical gRNAs in any of the folds. it makes no sense to report the performance over the 10 folds (the average I assume). The authors should report on the overlap of the gRNA (e.g. hamming distance) in the validation sets and show that this strategy do not lead to near identical gRNAs in different validation sets and thereby overfitting. If there are paralogue genes in different validation sets this could be a potential issue.*

Response:

We now explicitly state in the methods (“cross-validation for machine learning methods”) that our 10-fold cross-validation involved holding out one tenth of the genes for testing, and training on the other nine tenth of genes. There was no overlap between any test fold, i.e. each gene and guide falls in a test fold exactly once. We report the median Spearman correlation between predicted efficiency and measured depletion (calculated within each gene) across all held-out genes. We also now report the Hamming distance as suggested by the reviewer, with a minimum Hamming distance of 7 between test folds and average of 21 for our guide efficiency cross-validation. Between our training data and the purine genes used in our independent validation screen, the minimum Hamming distance was also 7 and the average 21.

----------------------------------------------------------------------------

*In relation to this, the size and selection of held out dataset for testing of 10-fold cross validation seem not to be described anywhere. What are the sizes of the validation sets and which genes AND gRNAs are in each of these sets. The authors should not only present this, but also justify that their specific split is representative, i.e., could the performance change drastically if they ended up with another split by the same criteria?*

Response:

We now explicitly state the size of each held out test fold in the methods (either 30 or 31 genes, targeted by between 624 and 806 guides). We have also explicitly defined the test and train guide sets in a new table S2. We have repeated the cross-validation procedure with different gene splits using two different seeds for randomization, and we see no qualitative differences in the results (results in table S7).

----------------------------------------------------------------------------

*It is unclear which gRNAs have been used for the independent test as sequences in both directions are given in Table S16. Also appear to be missing are sequence context of the targets and other inputs used for the model evaluation of these guides. The authors should clarify this.*

*It is also unclear why both strands seems to targeted, especially if NGG pam is needed*

*SG317 SG_sgRNA_degfp_ML1_F tagtAACGCCAGTGAAAAGCTCCA*

*SG318 SG_sgRNA_degfp_ML1_R aaacTGGAGCTTTTCACTGGCGTT*

*After some efforts, the upper case sequence appears to be the guide with _F matching the synthetic GFP gene. The test set is from Table S16, training data is from github https://github.com/BarquistLab/CRISPRi_guide_efficiency_bacteria.*

*This needs to be explained more carefully in the manuscript. Also it would be helpful if the authors explicitly point to github in this context.*

Response:

Table S17 (previously S16) “oligos” contains the oligonucleotide sequences used for cloning our expression constructs, explaining why there are both forward and reverse sequences. The complete guide sequences and context were given in Table S13 (previously Table S12) “purine_screen_guides”. In addition to the descriptions of the supplementary table previously provided in the first tab of the excel workbook, we have now also included Supplementary Table descriptions in the methods section of the manuscript as well, and explicit references to the Supplementary Tables where appropriate.

----------------------------------------------------------------------------

*Upon diving in to the data a bit of similarity to between training data and test data:*

*test training data from github*

*16 CGCTCCTGGACGTAGCCTTC CGCTTCTGTTCGTAGCTTTC*

*16 CGTCGCCGTCCAGCTCGACC TGTCGCCGCCCAGCTCGATT*

*16 CTTCAGGGTCAGCTTGCCGT CTTCCGGTGCAGCTTGCAGT*

*17 GACGTAGCCTTCGGGCATGG GACATAGCCTTCGGGAATAG*

*The authors are probably ok in overlap between test and training set, but it should be reported. What remain is that the authors justify why gRNAs on these genes are representative to serve as independent evaluation.*

Response:

We have now clarified in the text that the purine biosynthesis genes were chosen for validation as they are non-essential in rich medium, and thus not part of our training data that only included essential genes.

----------------------------------------------------------------------------

*The authors state that they "re-implement" CRISPRon. Since this is not actual CRISPRon method and since are profound differences (see the following) it is misleading and confusing to call it CRISPRon. The authors should call it some else and then when first time mentioned state precisely which parts are inspired by CRISPRon and which parts are not.*

Response:

We have moved the deep learning section to a supplementary note, as it was not a major point of the manuscript. We have revised the text so as to be clear that this is a reimplementation of the CRISPRon CGx model.

----------------------------------------------------------------------------

*In the scan of meta parameters for CNN and "CRISPRon" the authors should mention these, including batch size and learning rates. They should also point what is the same and difference for CRISPRon.*

Response:

We chose a batch size of 32, as our training dataset is quite small. We chose a learning rate of 0.001, as it is the default parameter for Adam. We have now additionally tried a batch size of 500 and learning rate of 0.0001 as used in the CRISPRon paper, resulting in similar performance (now shown in Table S7).

We also now specify that the main difference between our implementation and the CRISPRon implementation is in the choice of non-sequential features concatenated to the output of the convolutional subnetwork. This is mentioned in both the supplementary note and relevant methods section.

----------------------------------------------------------------------------

*It seems that the authors re-implementation of CRISPRon is not exact if the do not use the same architecture is as CRISPRon. The authors state (p15, 26-30): "we reimplemented and tested the state-of-the-art deep learning architecture used for predicting Cas9 gene editing efficiency by CRISPRon (Xiang et al., 2021), only trained using our CRISPRi data." However, they do not present how the resulting binding energy Delta GB is fed into the network. The authors need to explain this carefully.*

*Furthermore, it seems that the Yu et al, do not even have Delta GB as a single parameter and that "only" a hybridization energy and a gRNA spacer self-folding is included, but separately. And hence the authors seem to ignore the DNA opening energy. The authors should elaborate on what they precisely do and explain why their re-implementation ignores the opening energy.*

*The authors calculate DNA-gRNA hybridization energy from the Vienna package(p6, 11-12). As I understand Xiang their DNA-gRNA hybridization energy includes weights on each stacked pair corresponding to influence of the protein. This is apparently not the case for the authors re-implementation. Again, the authors need to elaborate and argue why their version of hybridization energy is more suitable.*

*For the SHAP analysis, I wonder if the authors compared to the one by Xiang et al, which showed that Delta GB is the most important feature SHAP and Gini importance. Can the authors comment on this?*

Response:

We would like to thank the reviewer for pointing out that we did not use the Delta GB parameter. We previously did not find thermodynamic features to have a large impact on efficiency predictions (e.g. they are not top features in Figure 3), so we did not investigate this as deeply as we might have. We have now tested Delta GB as a parameter in our MERF model and our CRISPRon-inspired deep learning model, finding that it does not improve performance, illustrated in Figure S8. It is unclear why thermodynamic features are less important for bacterial CRISPRi as compared to Cas9 genome editing.

----------------------------------------------------------------------------

*For the SHAP analysis on Fig 3A. The +1 PAM favoring efficient gRNAs was recently described by Corsi et al., 2022, Nat comm and the authors comment on that this already is known.*

Response:

We thank the reviewer for pointing this out. We have now added appropriate references for the +1G.

----------------------------------------------------------------------------

*There are what appear as as weird features in Table S3 including:*

*features Random Forest model trained with E75Rousset*

*gene_expression_max 0.782893970297067*

*ess_gene_operon 0.490418806310321*

*gene_GC_content 0.293801473999851*

*gene_expression_min 0.194561228228971*

*gene_length 0.237718945757704*

*geneid 0.174114175877214*

*distance_operon 0.151180464424676*

*dist_start_codon 0.147285478273062*

*dist_start_codon_perc 0.120338853153561*

*dataset 0*

*Although many of these seemingly have been removed to subsequent SHAP tables (S7 and S8) they should never have been used in the first place.*

Response:

The SHAP values in S3 are for our depletion model described in the first two sections of the manuscript, illustrated in Figure 1C. These are meant to illustrate the large effect gene features have on prediction of depletion (and importantly are not used to predict guide efficiency).

----------------------------------------------------------------------------

*More elaborate:*

*I am aware that the authors define what they refer to as call minimum and maximum expression values (p6, 34-37), but it is unclear precisely how they are defined.*

Response:

This is described in the Methods in the section “Feature Engineering” (pg 29):

“Minimal or maximal expression levels were calculated across the range of ODs until the growth phase when cells were collected in each CRISPRi screen: OD 1.4 for the Wang dataset, and all ODs for the Rousset and Cui datasets.”

We have now added an explicit reference to this section of the methods in the text.

----------------------------------------------------------------------------

*Do these minimum and maximum expression values even make sense when making prediction on an external data, where e.g. the expression is in another range? The authors should elaborate.*

Response:

As described at the beginning of our response, and now illustrated in figure 2A, gene features like expression are not used in making predictions with the fixed effect model.

----------------------------------------------------------------------------

*I am also concerned of the usage of the absolute position in the gene can create biases, since disruption closer to the 5' end will more potentially favor shorter genes and learn to distinguish short from long genes. The authors also show such bias in Fig 4E. Given the authors work with a small genome (compared to Eurakyote) the effect might be minor. Nonetheless and in spite that it sadly has been used on several instances in the literature, this feature should not be used. The authors remove this feature then report the performance again.*

Response:

The inclusion of this parameter was inspired by experimental work on CRISPRi. In particular the original Qi et al. 2013 paper introducing CRISPRi claimed that silencing efficiency depended in a linear fashion on distance from the start codon based on guides targeting two model genes. This assumption has been commonly adopted in the bacterial CRISPRi community, for instance the Wang et al. data used in this paper preferentially targeted close to the start codon, and the paper describing the Pasteur model only predicted guide efficiency within the first 100 bases of a gene annotation. In our saturating validation screen, we find that, contrary to these expectations, guides deep within the coding sequence can lead to effective depletion.

We have previously tested removing distance features (Figure S6EF&G, previously Figure S6B), and found that this dramatically reduces model performance. We have now additionally examined the effects of the distance features on efficiency predictions (Figure S5). It appears that the main impact of these features is on guides targeting a small window (~60 bases) near the start codon, and we speculate this may be associated with blocking polymerase binding at transcription start sites or preventing the first stages of transcriptional elongation, though the poor annotation of promoters in bacterial genomes precludes a robust analysis of this. We see no clear evidence that the distance features are learning to distinguish genes of different length, as the effect of distance outside this window are fairly small and roughly constant across typical gene lengths (Figure S5A).

We would also note that the panels in 4E show experimental measurements for each guide within each gene rather than model predictions and it is unclear how this shows bias in our model.

----------------------------------------------------------------------------

*Similar concerns can be raised for "gene length", "distance_operon" etc…*

Response:

As noted above, gene features are not used in making predictions with the fixed effect model but are meant to remove the effect of these potential confounders when extracting guide efficiency from depletion values across genes.

----------------------------------------------------------------------------

*What is potentially even bigger concerns is the authors use of "geneid" as a feature. Why would one what to label different gRNAs by the gene they come from? How would the authors in any meaningful way instruct a user that comes with an external data set, or simply just want to make a prediction on a specific gRNA?*

Response:

As described in our response to Reviewer 1, gene ID is used as a cluster label in our mixed effect model to remove the effect of the target gene when training our predictor of guide efficiency. It is not itself a predictor. Gene ID does not need to be supplied when making predictions on new guides with the fixed-effect model.

----------------------------------------------------------------------------

*Similar concern for "dataset", which I do not see clearly defined.*

Response:

“Dataset” is a categorical variable indicating which dataset a measurement came from for use in the random effect model and provides a dataset-specific offset. This was previously described in Table S1, but we have now added a direct reference to this from the Methods section.

----------------------------------------------------------------------------

*Minor:*

*When clicking on references I end up at unreadable links.*

Response:

Our apologies; this was an artifact of our reference manager. We have now removed the hyperlinks.

**Second round of review**

**Reviewer 1**

In response to my previous review, part of the answers are not fully satisfied.

Q1:

Satisfied.

Q2:

Satisfied.

Q3:

Gene expression is a secondary feature derived from primary features originated from sequence or environment conditions.

A high expression gene is likely to become sensitive towards CRISPRi operators, which seems obvious. Therefore, the utilization logic is rather weird, because one has to perform a RNA-seq experiment in order to get the gene expression value for designing the guide RNAs for the CRISPRi experiment.

In my opinion, the authors should redo the feature engineering part with usage of primary features based on sequence and cellular conditions.

Q4:

The authors did not explore deep learning method in depth. They claimed that data availability is the limitation. As far as I know, there exists plunty of regularization methods in deep learning, and the authors should explore some instead of simply ignoring it.

Q5:

Once more, the authors ignored the issue, yet just moved the unconvincing results from main text to supplementary part, which cannot be satisfied. Because given unconvincing results, they should improve the model and present a convincing results.

Q6:

Same as Q5, the result is unconvinced, so the authors should improve the model instead of explaining the reason why the model is not good.

**Reviewer 3**

The manuscript, "Improved prediction of bacterial CRISPRi guide efficiency from depletion screens through mixed-effect modeling and data integration" by Yu et al presents a mixed-effect random forest classifier that employs both gRNA features and gene features to predict gRNA efficiency in bacteria. The authors observe that integrating multiple datasets through data fusion enhances prediction performance, as do the inclusion of gene features.

However, the manuscript lacks clarity on the specific factors driving the predictions, which could potentially limit the reproducibility and interpretability of the results. Additionally, the extent of the model's generalizability to other biological systems remains uncertain and requires further exploration and validation. Furthermore, the paper's lack of statistical rigor is a concern. A more thorough explanation and justification of the statistical methods used, along with robust testing for significance and appropriate controls, are necessary to ensure the validity and reliability of the findings.

Concerns:

- It remains uncertain whether ML methods developed for other systems would also be successful in the context of bacteria. While the authors have chosen not to explore alternative methods in this study, it is essential to acknowledge that the state-of-the-art has not been established specifically for bacteria. The MERF model can be regarded as an improvement over the only other existing method, the Pasteur model, but definitive statements about the state-of-the-art should be avoided due to the absence of comprehensive research in this particular domain.

- Data fusion was demonstrated to be the major contributor to the observed performance gains, particularly with the Pasteur model. However, the potential benefits of incorporating additional features, such as gene features, into the Pasteur model remain uncertain. Notably, the concept of data fusion itself is not a novel proposal, as it has previously been shown to enhance the prediction of gRNA efficiency in another study [19].

- The additional claims made in the modeling process, including the identification of beneficial features, factors influencing performance, and model interpretability, are supported by limited anecdotal evidence. To enhance the rigor and establish more generalizable insights, conducting better-controlled experiments is essential. These improved control experiments will enable the researchers to identify and validate additional insights that can be reliably generalized beyond the specific context of this study.

- The results obtained from the SHAP analysis in this study appear to be suggestive, rather than definitive. To rigorously evaluate the predictive power of the features identified through SHAP analysis, the authors should perform a dedicated retraining of the models using only these SHAP-based features. This retraining will enable a quantitative assessment of the predictive performance of the selected features, such as measuring the variance explained by the models based on the SHAP-informed feature set. By doing so, the authors can strengthen their findings and provide more robust evidence for the importance and utility of these features in the predictive models.

- Including additional baselines, such as random forest and gradient-boosted trees (xgboost) using the same set of features, would provide valuable insights into the benefits of the MERF model. By comparing the performance of these baselines with MERF, it becomes possible to distinguish whether the advantages of MERF arise primarily from handling non-linearity or from its ability to decompose mixed effects.

- The description of the automated ML process in the methods section lacks clarity, as it only provides a list of sklearn commands. The reader should not be required to refer to code documentation or read the implementation details to understand the steps performed. A more detailed and explicit explanation of the automated ML procedure should be provided.

- Error bars should be shown across the k-folds. Also statistical tests can be performed to show whether the differences are significant?

**Authors Response**

**Point-by-point responses to the reviewers’ comments:**

We thank the reviewers for their valuable comments, and the editor for giving us the opportunity to revise the manuscript. We have introduced the following major additions in response to the reviewers’ comments:

-Feature importance validation: We validated our SHAP-derived importance through computational experiments where we dropped one feature at a time during training. The results of this show that our top ten important features all contribute to model performance.

-We have now included p-values for all quantitative claims in the manuscript. This has not led to any changes in our claims or conclusions.

-Inclusion of a Random Forest Baseline Model: We now include evaluation of a random forest trained on activity scores (as defined in figure S4) as a comparator for our MERF model. This analysis indicates the ability to handle non-linear feature interactions may be the primary driver of improved performance for our approach over the Pasteur model.

All changes to the text have been highlighted in red in the revised manuscript.
—----------------

*Reviewer #1: In response to my previous review, part of the answers are not fully satisfied.

Q1:
Satisfied.

Q2:
Satisfied.

Q3:
Gene expression is a secondary feature derived from primary features originated from sequence or environment conditions.
A high expression gene is likely to become sensitive towards CRISPRi operators, which seems obvious. Therefore, the utilization logic is rather weird, because one has to perform a RNA-seq experiment in order to get the gene expression value for designing the guide RNAs for the CRISPRi experiment.
In my opinion, the authors should redo the feature engineering part with usage of primary features based on sequence and cellular conditions.*

Response:
The reviewer raises an important point regarding the requirement for RNA-seq data to train the MERF model. However, we disagree with the reviewer that it is obvious that high expression should increase gene sensitivity to CRISPRi silencing. To our knowledge, this effect has not previously been reported in the literature and has implications beyond training our model of CRISPR guide efficiency. If anything, one would expect low-expressed genes should be more sensitive to silencing, as fewer RNA polymerases would lend to bound dCas9 serving as a stronger roadblock.

We would also like to clarify that the RNA-seq data is only used to train the initial model, and is not a requirement for predicting guide efficiency with the trained model. As model training for a new organism or CRISPRi system would require performing and sequencing a genome-wide screen, we do not believe performing an additional RNA-seq experiment is a major barrier to adoption, and our results suggest that RNA-seq data would be helpful in optimizing future guide designs with any method. Performing RNA-seq would also be non-obvious to those in the field given the general focus on sequence-specific features rather than gene-specific features.

Nevertheless, we have already addressed the reviewer’s current suggestion to redo all feature engineering based solely on primary sequence features in our previous response to this point, to quote: “We have additionally tested using the codon adaptation index (CAI), a proxy for gene expression based solely on codon usage patterns in the genome sequence, as an alternative to including actual expression data for training. We achieve similar performance using CAI in place of expression data (Figure S7GH&I), further indicating that the performance of our model is not dependent on having access to expression information specific to assay conditions.” This was also briefly mentioned in the discussion of the previous version of the manuscript.
—----------------------

*Q4:
The authors did not explore deep learning method in depth. They claimed that data availability is the limitation. As far as I know, there exists plunty of regularization methods in deep learning, and the authors should explore some instead of simply ignoring it.*

Response:
We have implemented two deep learning methods, including a recent architecture developed to predict Cas9 genome editing in eukaryotes, and neither improved on the performance of the MERF model. While we acknowledge in the text that it may be possible to improve on these results through an extensive exploration of different methods and architectures, we do not believe this is guaranteed to work and would furthermore not be feasible within the time frame of a revision.

We think it is unlikely that regularization alone would solve the data limitation problem. We are not the first to see a dependence of the performance of deep learning guide activity prediction algorithms on training dataset size; for instance Kim et al, Nature Biotech 2018 (Figure 1B) show it took between 2000 and 4000 training samples before deep learning approaches clearly outperformed linear regression for predicting Cpf1 genome editing efficiency , using a direct read-out of guide efficiency. Additionally, there is work showing that tree-based methods generally outperform deep learning on small-to-medium-sized tabular data, similar to the training data we have available here (Grinsztajn, Oyallon and Varoquaux, “Why do tree-based models still outperform deep learning on tabular data?” arXiv 2022), and that developing deep learning methods that are effective for such data remains an open problem in the machine learning field.
—--------------------------------------
 *Q5:
Once more, the authors ignored the issue, yet just moved the unconvincing results from main text to supplementary part, which cannot be satisfied. Because given unconvincing results, they should improve the model and present a convincing results.*

Response:
We would like to emphasize that the results in figure S6 show that the MERF outperforms the Pasteur model in two out of three low-throughput validation experiments. While it would likely be possible to optimize our model to also outperform the Pasteur on the Miller assay dataset in S6C, we believe the practice of modifying the model specifically to fit a small independent validation dataset is likely to lead to overfitting and reduced generalizability.
—---------------------------------------

*Q6:
Same as Q5, the result is unconvinced, so the authors should improve the model instead of explaining the reason why the model is not good.*

Response:
We disagree with the reviewer that the model is “not good”, as we have clearly shown that our final model improves on previous approaches to predicting CRISRPi guide efficiency. In particular, we have shown that the MERF model trained on three datasets clearly outperforms the previous Pasteur model in both cross-validation and in predicting depletion in an independent saturating screen. We have also undertaken significant work during revision to show that retraining the Pasteur model alone is not sufficient to achieve the performance of our MERF model. We do not believe the relative performance of the MERF model trained on a single dataset affects these conclusions.
—----------------------------------------------


*Reviewer #3: The manuscript, "Improved prediction of bacterial CRISPRi guide efficiency from depletion screens through mixed-effect modeling and data integration" by Yu et al presents a mixed-effect random forest classifier that employs both gRNA features and gene features to predict gRNA efficiency in bacteria. The authors observe that integrating multiple datasets through data fusion enhances prediction performance, as do the inclusion of gene features.

However, the manuscript lacks clarity on the specific factors driving the predictions, which could potentially limit the reproducibility and interpretability of the results. Additionally, the extent of the model's generalizability to other biological systems remains uncertain and requires further exploration and validation. Furthermore, the paper's lack of statistical rigor is a concern. A more thorough explanation and justification of the statistical methods used, along with robust testing for significance and appropriate controls, are necessary to ensure the validity and reliability of the findings.

Concerns:
- It remains uncertain whether ML methods developed for other systems would also be successful in the context of bacteria. While the authors have chosen not to explore alternative methods in this study, it is essential to acknowledge that the state-of-the-art has not been established specifically for bacteria. The MERF model can be regarded as an improvement over the only other existing method, the Pasteur model, but definitive statements about the state-of-the-art should be avoided due to the absence of comprehensive research in this particular domain.*

Response:
We would like to point out that we have in fact shown that four methods for predicting CRISPR guide efficiency for eukaryotic genome editing are not directly applicable to predicting CRISPRi guide efficiency in bacteria (Figure S7D). While this might be expected, as these methods have not been specifically trained for bacterial CRISPRi, many in the field would assume that the determinants of Cas9 binding are similar enough across organisms and assays that such tools might work. We show definitively that they do not.

We would also like to emphasize that these methods are generally not easily adaptable to the problem of predicting guide efficiency for CRISPRi from depletion screens through retraining. The training data that has generally been used for eukaryotic genome editing applications is a direct readout of editing efficiency by e.g. genomic sequencing of edited clones. In our case, we only have an indirect measurement of efficiency, that is depletion of strains expressing a particular guide RNA, which is the result of a combination of the fitness effect from gene silencing along with the efficiency of gene silencing itself (illustrated in Figure 1A). In the first section of the manuscript, we show that the gene effect dominates over features manipulable in guide design. This significantly complicates both training and evaluation, as the true training target (guide efficiency) is unknown, and led us to adopt the MERF approach to segregate these mixed effects within the model itself.

Regardless, we have removed all uses of the phrase “state-of-the-art” to describe previous methods for predicting bacterial CRISPRi guide efficiency from the manuscript.
—--------------------------------------------

*- Data fusion was demonstrated to be the major contributor to the observed performance gains, particularly with the Pasteur model. However, the potential benefits of incorporating additional features, such as gene features, into the Pasteur model remain uncertain. Notably, the concept of data fusion itself is not a novel proposal, as it has previously been shown to enhance the prediction of gRNA efficiency in another study [19].*

Response:
We would like to clarify that it is not possible to include gene features in the Pasteur model and still be able to predict the efficiency of e.g. a guide targeting a new or held-out gene, due to conflation of gene and guide effects. However, we did retrain the Pasteur model using all of our guide features, and this led to reduced performance in our validation screen compared to the retrained Pasteur using only the original feature set (rho=0.505 v 0.569). We have not included this result in our revision, as we do not believe it significantly affects any of our conclusions.
—------------------------------------------------

*- The additional claims made in the modeling process, including the identification of beneficial features, factors influencing performance, and model interpretability, are supported by limited anecdotal evidence. To enhance the rigor and establish more generalizable insights, conducting better-controlled experiments is essential. These improved control experiments will enable the researchers to identify and validate additional insights that can be reliably generalized beyond the specific context of this study.*

Response:
We appreciate the reviewer's suggestion. We would like to emphasize that the selection of relevant features, model types, and the method for model interpretation in our study was not arbitrary but based on rigorous considerations. We believe this exceeds the general standard applied in the CRISPR/Cas efficiency prediction literature, and certainly extends what has previously been shown for bacterial CRISPRi.

-First, we thoroughly explored biological features that could affect strain depletion in CRISPRi screens, which we rigorously evaluated by systematic testing where each feature's relevance was determined by examining the performance of sequentially more complex predictive models (Figure 1B).

-Secondly, in addition to using automated machine learning (autoML), we thoroughly explored various common machine learning models as a comparative exercise, aiming to verify autoML's performance and robustness (Figure S3).

-Finally, for model interpretation, we adopted SHAP, a method that provides a robust approximation to the effects of leaving a single feature out.

In response to the reviewer’s comments we have performed a number of additional computational experiments, including testing different training schemes and performing leave-one-feature out experiments for our top important features as determined by SHAP, discussed in detail below. We hope these modifications address the reviewer's concern adequately.
—--------------------------------------------


*- The results obtained from the SHAP analysis in this study appear to be suggestive, rather than definitive. To rigorously evaluate the predictive power of the features identified through SHAP analysis, the authors should perform a dedicated retraining of the models using only these SHAP-based features. This retraining will enable a quantitative assessment of the predictive performance of the selected features, such as measuring the variance explained by the models based on the SHAP-informed feature set. By doing so, the authors can strengthen their findings and provide more robust evidence for the importance and utility of these features in the predictive models.*

Response:
We appreciate the reviewer’s point that SHAP values are only an approximation of the effects of removing a given feature. We would like to point out that directly measuring the variance explained by the models on the target variable (guide efficiency) is nontrivial, as the true target is unknown. However, we have addressed the reviewer’s point by dropping predicted important features and observing the effects on the Spearman correlation in our validation screen.

To illustrate that the features selected by SHAP analysis affect model performance, we trained a series of models dropping a single residue feature (Figure S7B). We find that removing each of these features leads to significant decreases in performance on our validation data as measured by the median Spearman correlation; this is in addition to our previous results illustrating the impact of distance features on model performance (Figure S7G). These results are now discussed on page 14. We believe that this shows conclusively that the features selected as important by SHAP do in fact affect model performance.

As the reviewer suggested, we also trained a model only using the top ten features identified by SHAP analysis. This model performed worse than our full model, indicating that additional features besides the top ten contribute to model performance. We have not included this result in the current version of the manuscript as we do not claim that the top ten features reported by SHAP are the only important features for guide design.
—---------------------------------------------

*- Including additional baselines, such as random forest and gradient-boosted trees (xgboost) using the same set of features, would provide valuable insights into the benefits of the MERF model. By comparing the performance of these baselines with MERF, it becomes possible to distinguish whether the advantages of MERF arise primarily from handling non-linearity or from its ability to decompose mixed effects.*

Response:
The reviewer makes a good point, and such a baseline random forest model was included in our initial submission but removed after the first round of review. We now include a comparison between MERF and a new baseline random forest model in Figure S7C. It should be noted this model is trained on activity scores as defined in Figure S4A and used for the Pasteur model, as it is not possible to directly predict guide efficiency from depletion scores without considering mixed effects in some way. The performance of the baseline RF is slightly, but not significantly, worse than the MERF which is perhaps not surprising given the strong correlation we previously reported between calculated activity scores and the predicted random effects from the MERF model in Figure S4E. These results are discussed on page 14.
—--------------------------------------

*- The description of the automated ML process in the methods section lacks clarity, as it only provides a list of sklearn commands. The reader should not be required to refer to code documentation or read the implementation details to understand the steps performed. A more detailed and explicit explanation of the automated ML procedure should be provided.*

Response:
We have expanded our description of the automated machine learning process in the methods to better explain the meaning of the chosen parameters (pages 32-33).
—----------------------------------------

*- Error bars should be shown across the k-folds. Also statistical tests can be performed to show whether the differences are significant?*

Response:
We have now included a boxplot showing the cross-validation results for the MERF and Pasteur models (Figure S4F). We have now also included p-values based on paired t-tests for all quantitative claims in the text. This has not led to any changes in our individual claims or conclusions.
—-------------------------------------------

**Third round of review**

**Reviewer 3**

The authors have adequately satisfied my concerns. The SHAP-based feature ablation and improved statistics throughout the manuscript makes this a more rigorous study.
